# The Incidence of Japanese Encephalitis in Taiwan—A Population-Based Study

**DOI:** 10.1371/journal.pntd.0003030

**Published:** 2014-07-24

**Authors:** Li-Ching Hsu, Yu-Ju Chen, Feng-Kuang Hsu, Jyh-Hsiung Huang, Chi-Ming Chang, Pesus Chou, I-Feng Lin, Feng-Yee Chang

**Affiliations:** 1 Center for Research, Diagnostics and Vaccine Development, Centers for Disease Control, Ministry of Health and Welfare, Taipei, Taiwan, Republic of China; 2 Institute of Public Health, School of Medicine, National Yang-Ming University, Taipei, Taiwan, Republic of China; 3 Epidemic Intelligence Center, Centers for Disease Control, Ministry of Health and Welfare, Taipei, Taiwan, Republic of China; 4 Community Medicine Research Center, National Yang-Ming University, Taipei, Taiwan, Republic of China; 5 Centers for Disease Control, Ministry of Health and Welfare, Taipei, Taiwan, Republic of China; 6 Division of Infectious Diseases and Tropical Medicine, Department of Internal Medicine, Tri-Service General Hospital, National Defense Medical Center, Taipei, Taiwan, Republic of China; U.S. Naval Medical Research Unit No. 2, Indonesia

## Abstract

**Background:**

A mass Japanese encephalitis (JE) vaccination program targeting children was launched in Taiwan in 1968, and the number of pediatric JE cases substantially decreased thereafter. The aim of this study was to elucidate the long-term trend of JE incidence, and to investigate the age-specific seroprevalence of JE-neutralizing antibodies.

**Methodology/Principal Findings:**

A total of 2,948 laboratory-confirmed JE cases that occurred between 1966 and 2012 were analyzed using a mandatory notification system managed by the Centers for Disease Control, Taiwan. A total of 6,594 randomly-sampled serum specimens obtained in a nationwide population-based survey in 2002 were analyzed to estimate the seroprevalence of JE-neutralizing antibodies in the general population. The average annual JE incidence rate of the group aged 30 years and older was 0.167 cases per 100,000 people between 2001 and 2012, which was higher than the 0.052 cases per 100,000 people among those aged under 30 years. These seroepidemiological findings indicate that the cohort born between 1963 and 1975, who generally received two or three doses of the vaccine and were administered the last booster dose more than 20 years ago, exhibited the lowest positive rate of JE-neutralizing antibodies (54%). The highest and second highest antibody rates were observed, respectively, in the oldest unvaccinated cohort (86%) and in the youngest cohort born between 1981 and 1986, who received four doses 10–15 years ago (74%).

**Conclusion/Significance:**

Over the past decade, the main age group of the confirmed JE cases in Taiwan shifted from young children to adults over 30 years of age. People who were born between 1963 and 1975 exhibited the lowest seroprevalence of JE-neutralizing antibodies. Thus, the key issue for JE control in Taiwan is to reduce adult JE cases through a cost-effective analysis of various immunization strategies.

## Author Summary

JE is one of the major public health problems in Asian and the Western Pacific regions, and most cases occur in children under the age of 14 years. A JE virus infection can cause severe sequelae such as an impairment of language ability, cognitive ability, or movement. Because humans are a dead-end host of the JE virus, the disease cannot be transmitted among people. Vaccination is currently the most effective method for preventing JE, and children in most endemic areas are vaccinated. After decades of mass vaccination, the number of confirmed JE cases has considerably declined in Taiwan, Japan, and South Korea. Most JE cases have occurred in adults rather than children in these countries, thus, the disease must be controlled by reducing the number of adult JE cases. Therefore, a prevention policy for the adult and elderly population should be implemented in the near future.

## Introduction

Japanese encephalitis virus (JEV) infection is a major public health problem across Asia and the Western Pacific Region [1], [2], and has recently spread to new territories such as Papua New Guinea and Northern Australia [3]–[5]. The World Health Organization estimates that approximately 67,900 cases of JE occur worldwide every year, 75% of which affect children under 14 years of age [4]. Many of these patients suffer from severe symptoms, and the case-fatality rate is 20% to 30%. Furthermore, 30% to 50% of the survivors suffer from permanent neurological or psychiatric sequelae [5]–[13]. No specific antiviral treatments are currently available, and vaccination remains the most effective protection against JEV infection.

JE is a mosquito-borne zoonotic infectious disease, and JEV belongs to the genus Flavivirus of the family Flaviviridae. JEV can be transmitted by vector mosquitoes, with *Culex tritaeniorhynchus* being the primary vector [14] in Taiwan; pigs are the main amplifying hosts, and humans are dead-end hosts. Outbreaks typically occur during summer in Taiwan, peaking between May and August [15], [16].

A comprehensive vaccination campaign for children has been implemented in Taiwan since 1968, when a peak of 200 JE cases was reached, and the number of confirmed cases has declined each year since then. After the introduction of the JE vaccination policy across Taiwan, JE cases shifted from children to adults [13], and this pattern has also been observed in neighboring countries such as Japan and South Korea [11], [17], [18]. Several studies on the seroprevalence of JE were conducted in Taiwan in the 1990s; however, these studies were confined to small local areas, and their definition of seropositivity of antibodies was inconsistent [19]–[21]. The purpose of this study was to elucidate the long-term trend in JE incidence over the past 4 decades by using population surveillance data, and to investigate the prevalence of serum neutralization antibodies, based on a nationwide large-scale sample in Taiwan.

## Materials and Methods

### Ethics Statement

Institutional review board approval was obtained from Taiwan Disease Control, Ministry of Health and Welfare. Data of JE laboratory-confirmed cases between 1966 and 2012 were collected from the Taiwan Centers for Disease Control (CDC), and all the JE cases were anonymized and analyzed as secondary data. Serum samples were obtained from the Taiwanese Survey on the Prevalence of Hyperglycemia, Hyperlipidemia, and Hypertension (TwSHHH), funded by the Health Promotion Administration, Ministry of Health and Welfare, Taiwan in 2002. All the participants provided written informed consent before blood samples were collected, and all the serum samples were anonymized. In signing the written informed consent, all the TwSHHH participants also agreed that their remaining serum specimens could be used for other hyperglycemia, hyperlipidemia, and hypertension studies, as well as for public-health-related research.

### Japanese Encephalitis Surveillance

JE has been a reportable communicable disease in Taiwan since 1955, and physicians are required to report incidents by using a hospital-based passive reporting system. Laboratory testing methods were established in 1965 to examine reported cases, followed by the implementation of an active JE surveillance system in 1967. Health care providers must notify the health authorities of any case that meets the case definition by law [13], and should collect and send specimens to the central CDC laboratory of Taiwan for examination. In addition, the local health authorities are responsible for epidemic investigation and related tasks, such as vector control. All suspected cases are archived and centrally managed by the Taiwan CDC.

### Japanese Encephalitis Cases

According to a laboratory diagnosis, only two flaviviruses, JEV and the dengue virus, are known to be circulating in Taiwan. JEV is circulating nationwide, whereas the dengue virus is confined to Southern Taiwan. In 1998, the Taiwan CDC developed an E/M-specific capture IgM and IgG enzyme-linked immunosorbent assay (E/M-specific IgM/IgG ELISA) for JE and dengue fever (DF). However, antibodies against both JE and DF are screened using hemagglutination inhibition (HI) or an E/M-specific IgM/IgG ELISA, and the DF antibody test results are used as a negative control to reduce the probability of cross-reactivity. A long-term evaluation revealed that the E/M-specific IgM/IgG ELISA is highly sensitive and specific, and can effectively differentiate JEV from dengue virus infection [22]. Since 2001, acute phase serum obtained within 7 days and cerebrospinal fluid (CSF) of JE cases have been used for diagnosis through a real-time polymerase chain reaction (PCR) method; nevertheless, the E/M-specific IgM/IgG ELISA remains the primary method for diagnosis.

The JE laboratory confirmed that cases that met one of certain laboratory criteria were defined as being JE-confirmed cases. These criteria were: 1) an HI titer of the convalescent serum of ≥1∶160, and at least a four-fold rise between the HI titers of convalescent and acute sera; 2) an HI titer from a single serum of ≥1∶320; 3) an IgM positive serum was obtained using the ELISA test, or the IgG of the paired serum exhibited a four-fold increase; 4) a sample that exhibited a positive real-time PCR reaction; or 5) a sample that was positive to IFA staining after isolating the virus. In addition to meeting this case definition, JE cases are not confirmed until they have been positively diagnosed in a laboratory. This study comprised 2,948 cases out of the 2,954 cases confirmed between 1966 and 2012, excluding cases in six non-Taiwanese citizens.

The JE incidence rates of various age groups or birth cohorts were calculated using the 1966–2012 census population data obtained from the Department of Household Registration, Taiwan; these censuses are house-to-house and are conducted by the government every 10 years.

### Vaccination Histories

The Taiwanese government launched a comprehensive vaccination campaign against JE in the 1960s, during which all children younger than 3 years received two doses of JE vaccines. After 1974, the vaccination doses were increased to three with a booster dose administered one year after the two primary doses. After 1976, a fourth booster dose was administered to children during their first year at elementary school. After 1980, the target population for JE vaccination has mainly been children older than 15 months. The vaccination is administered in two doses separated by an interval of 2 weeks; it is administered before the epidemic season, which is from March to May, and this is followed by a booster dose one year later, and a final booster dose (the fourth dose) when the child enters the first year of elementary school.

### Seroprevalence of the Japanese-Encephalitis-Neutralizing Antibody

The serum samples used were obtained from the TwSHHH (2002) [23], [24]. The TwSHHH subjects were enrolled from the National Health Interview Survey (NHIS) in 2001, which used a multistage, stratified, and clustered sampling scheme that included 26,685 noninstitutionalized residents from 6,592 households in 1,648 communities in Taiwan. As part of the TwSHHH, half of the NHIS-sampled Household Registration List (3,296 households) was randomly selected from each stratum. Out of 10,292 eligible subjects (73.6% attendance rate), 7,578 agreed to participate in the TwSHHH, and provided informed consent; 6,602 of the subjects who agreed to provide a blood sample were aged over 16 years, and 6,594 serum specimens were collected and included in the present study. Urbanization was divided into three levels, urban, suburban, and rural areas, according to previous studies [25], [26].

### Plaque Reduction Neutralization Test for the Seroprevalence of Japanese Encephalitis

The plaque reduction neutralization test (PRNT) was used to detect the JE-neutralizing antibodies. The PRNT is the most frequently used and standardized method for detecting protective antibodies against the JE virus [27], [28]. In general, a PRNT50 titer of ≥1∶10 represents an effective protection [29], [30]. The JE vaccine strain that was used during the vaccination campaign was the Nakayama-NIH strain, and this strain was also used in the PRNT. The test sera were diluted (1∶10) in Dulbecco's Minimum Essential Medium (MEM) with 5% FCS after remaining inactive at 56°C for 30 minutes. An equal volume of JE virus solution, calculated to yield approximately 100 plaque-forming units (PFUs) per 0.1 mL, was added to each serum dilution, and incubated at 4°C overnight. The mixture was then added in duplicate to a monolayer of the cell line BHK-21 (Baby Hamster Kidney) in a 24-well plate. After adsorption for one hour at 37°C with 5% CO_2_, the monolayer in each well was overlaid with 0.5 mL of 1% methyl cellulose in MEM containing 5% FCS. After 3–5 days of incubation at 37°C with 5% CO_2_, the cells were stained with 0.1% Naphthol Blue Black, and washed with normal saline. The neutralizing antibody titer was pinpointed as the reciprocal of the serum dilution that inhibited 50% or more of the plaque formation compared with the virus control group.

### Statistical Analysis

The incidence rate of JE was calculated using the JE confirmed cases per 100,000 people at risk. A multiple logistic regression analysis was used to estimate the odds ratio and 95% confidence interval for each factor that was associated with the JE-neutralizing antibody. The dependent variable was the presence of a JE-neutralizing antibody in the individual: a value of 1 was assigned for the presence of the antibody, and 0 for the absence. The independent variables included sex, birth cohort (six cohorts represented by five dummy variables), and urbanity (three degrees represented by two dummy variables). The statistical significance level was set at 0.05. All computations were performed using the SAS 9.2 statistical software package (SAS Institute Inc., Cary, NC, USA).

## Results

### Incidence Rates of Japanese Encephalitis between 1966 and 2012

**Figure 1:**
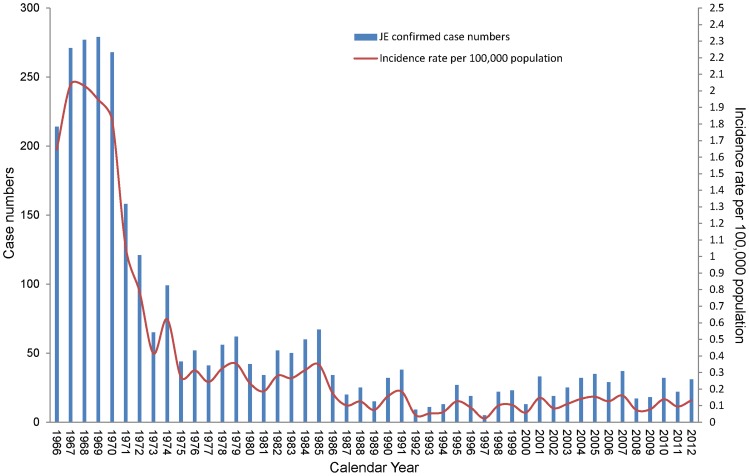
The cases number of Japanese Encephalitis and incidence rate in Taiwan during 1966–2012.

The annual incidence rates and number of confirmed JE cases from 1966 to 2012 are presented in Figure 1. In this study, 2,948 JE-confirmed cases were analyzed. The incidence rates of confirmed cases dropped rapidly after 1971. The lowest incidence rate occurred between 1992 and 1997, with a slight increase from 1998, followed by a stable state.

The highest incidence rates, ranging from 1.65 to 2.04 cases per 100,000 people, occurred between 1966 and 1970. The incidence rates reached more than 2.0 cases per 100,000 people in 1967 and 1968. Approximately 200 to 300 confirmed cases were detected in these 2 years, and the number of cases substantially declined thereafter. The incidence rate decreased slowly and steadily from 1975 to 1991, ranging from 0.1 to 0.35 (0.233 on average) cases per 100,000 people, with approximately 15 to 67 confirmed cases every year. The lowest incidence rates corresponded to the period between 1992 and 1997, with 0.126 to 0.023 (0.066 on average) cases per 100,000 people, or between 5 and 27 confirmed cases every year. After 1998, the incidence rates were between 0.161 and 0.058 (0.128 on average) cases per 100,000 people, with an annual number of confirmed cases in the range of 13 to 37 cases, and 26 cases on average.

The average annual incidence rate of JE cases was approximately 0.118 cases per 100,000 people between 2002 and 2012 (Table 1). Only three of the 297 confirmed cases corresponded to vaccinated people. Two of them received only one dose, and the other received three doses; 60% of the confirmed cases were male, and 61.4% of them were older than 40 years.

**Table 1:** Incidence rates and onset age distributions of Japanese Encephalitis confirmed cases in Taiwan, 2002–2012.

					Onset age in years, n(%)						
Calendar year	Confirmed cases, n	Incidence rate^a^	Male sex, n(%)	Vaccination history, ≥1 dose^b^	0–9	10–19	20–29	30–39	40–49	50–59	> = 60
2002	19	0.084	9(47.4)	0	0	2(10.5)	4(21.1)	2(10.5)	6(31.6)	4(21.1)	1(5.3)
2003	25	0.111	15(60)	0	1(4.0)	2(8)	7(28)	8(32)	5(20)	0	2(8)
2004	32	0.141	18(56.3)	0	0	1(3.1)	4(12.5)	7(21.9)	9(28.1)	9(28.1)	2(6.3)
2005	35	0.154	25(71.4)	0	0	2(5.7)	2(5.7)	10(28.6)	9(25.7)	8(22.9)	4(11.4)
2006	29	0.127	17(58.6)	0	0	1(3.4)	0	7(24.1)	8(27.6)	10(34.5)	3(10.3)
2007	37	0.161	19(51.4)	0	1(2.7)	1(2.7)	3(8.1)	9(24.3)	8(21.6)	9(24.3)	6(16.2)
2008	17	0.074	12(70.6)	2	1(5.9)	2(11.8)	2(11.8)	3(17.6)	8(47)	1(5.9)	0
2009	18	0.078	13(72.2)	0	0	1(5.6)	2(11.1)	1(5.6)	6(33.3)	3(16.7)	5(27.8)
2010	32	0.138	21(65.6)	0	1(3.1)	0	5(15.6)	5(15.6)	7(21.9)	9(28.1)	5(15.6)
2011	22	0.095	14(63.6)	0	1(4.5)	0	1(4.5)	7(31.8)	3(13.6)	6(27.3)	4(18.2)
2012	31	0.133	16(51.6)	1	0	0	3(9.7)	6(19.4)	9(29)	8(25.8)	5(16.1)
**Total**	**297**	**0.118**	**179(60.3)**	**3**	**5(1.7%)**	**12(4.0%)**	**33(11.1%)**	**65(21.9%)**	**78(26.3%)**	**67(22.6%)**	**37(12.5%)**

a The incidence rate is the number of JE confirmed cases per 100,000 population at risk.

b There were two JE confirmed cases have been received 1 dose of vaccine in 2008 and one has been vaccinated with 3 doses, in 2012.

### Age Distribution of Confirmed Japanese Encephalitis Cases

**Figure 2:**
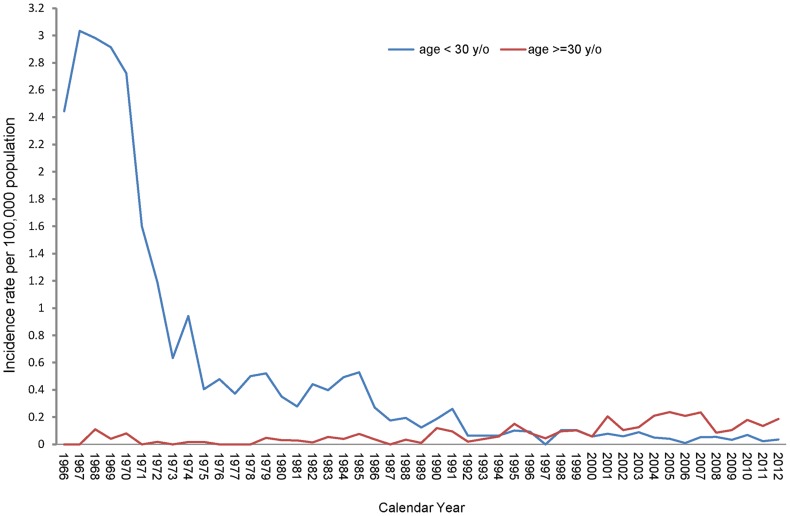
Age-specific incidence rates of Japanese Encephalitis during 1966–2012.

The JE incidence rates of various age groups of the Taiwanese population are displayed in Figure 2. The epidemic status of JE over the years can be approximately divided into three stages. Before 1991, the confirmed JE cases primarily occurred in residents aged 0–29 years, with incidence rates that ranged between 0.124 and 3.03 cases per 100,000 people. The highest incidence rate in the age group of 0–29 years occurred between 1966 and 1970 (2.44–3.03 cases per 100,000 people), and declined rapidly thereafter. The average incidence rates and number of confirmed cases were similar for both age groups (<30 and ≧30 years old) from 1992 to 2000, and they were also the lowest compared to any other period. However, after 2001, the incidence rates among people aged 30 years or older were higher than those of younger people (0.167 vs. 0.052 cases per 100,000 people on average).

### Japanese Encephalitis among Various Birth Cohorts

**Figure 3:**
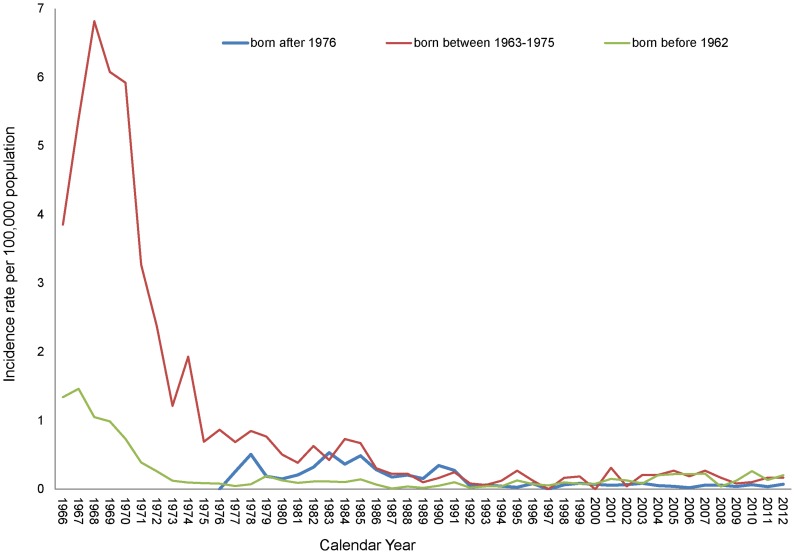
Japanese Encephalitis incidence rates among different birth cohorts during 1966–2012.

Figure 3 displays the incidence rates of JE across the three birth cohorts comprising people who were subjected to various vaccination schedules, namely those born before 1962, those born between 1963 and 1975, and those born after 1976. People born between 1963 and 1975 generally exhibit higher incidence rates than those of other groups, particularly during the period from 1967 to 1970 (from 5.4 to 6.82 cases per 100,000 people). The incidence rates were similar for the three birth cohorts from 1992 to 2002. Since 2004, the incidence rates of JE for those born between 1963 and 1975, or before 1962, have been slightly higher than those for people born after 1976.

### Age-Specific Seropositive Rates of Japanese-Encephalitis-Neutralizing Antibodies

**Figure 4:**
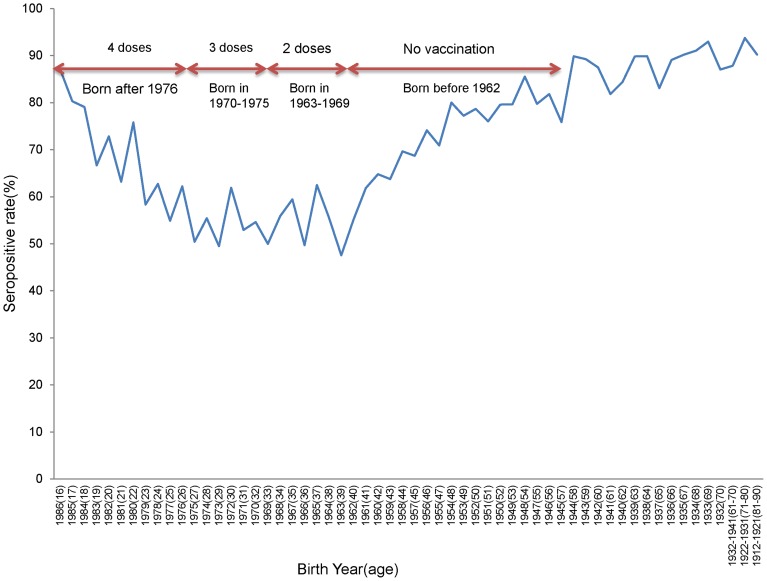
Seropositive rates of Japanese Encephalitis neutralizing antibody by birth year (age at blood drawn).

The overall positive rate of JE-neutralizing antibodies in participants aged over 16 years was 71% in 2002. The age-specific seropositive rates of JE neutralization in people over 16 years in 2002 are displayed in Figure 4. The seropositive rate decreased gradually from 87% in people born in 1986 to 62% in people born in 1976 (who received four doses of the JE vaccine). The seropositive rates remained low (approximately 50%–60%) in people born between 1963 and 1975 (who received two or three doses of the JE vaccine). The seropositive rates increased gradually from 70% in people born in 1958 to approximately 90% in people born after 1943. People born before 1962 were never vaccinated because the immunization program had not yet been introduced.

### Possible Factors that Affect the Level of Japanese-Encephalitis-Neutralizing Antibodies

To evaluate the influence of gender, birth cohort, and urbanization on the odds of presenting JE-neutralizing antibodies, a logistic regression model analysis was performed (Table 2). The results revealed that when the birth cohort and degree of urbanization were adjusted for, the odds of presenting JE-neutralizing antibodies were significantly higher in men than in women, with an odds ratio of 1.25 (95% CI = 1.12–1.40). Thus, the male JE seroprevalence was significantly higher than that of women.

**Table 2:** Multiple logistic regression analysis of factors associated with Japanese Encephalitis neutralizing antibody in Taiwan, 2002.^a^

		No. of positive/n	Positive rate(%)	OR(95%CI)	p-value
All subjects		4681/6594	71.0		
Gender	female	2363/3431	68.9	1(reference)	
	male	2318/3163	73.3	1.25(1.12–1.40)	<0.0001
Birth cohort	1981–1986 (received 4 doses)	526/710	74.1	1(reference)	
	1976–1980 (received 4 doses)	375/595	63.0	0.56(0.47–0.75)	<0.0001
	1970–1975 (received 3 doses)	369/676	54.6	0.42(0.33–0.53)	<0.0001
	1963–1969 (received 2 doses)	523/963	54.3	0.42(0.34–0.52)	<0.0001
	1953–1962 (No JE vaccine)	962/1409	68.3	0.77(0.63–0.94)	0.012
	1912–1952 (No JE vaccine)	1926/2241	85.9	2.19(1.78–2.69)	<0.0001
Urbanicity	urban	943/1458	64.7	1(reference)	
	suburban	3150/4360	72.2	1.52(1.34–1.74)	<0.0001
	rural	588/776	75.8	1.61(1.31–1.98)	<0.0001

a All variables were included gender, birth cohorts and urbanicity.

The JE-neutralizing antibody positive rates were 74%, 63%, 55%, 54%, 68%, and 86% from the youngest to the oldest cohorts. When the gender and degree of urbanization were adjusted for, the birth cohorts of 1976–1980, 1970–75, 1963–1969, and 1953–1962 were less likely to test positive to JE-neutralizing antibodies than those born between 1981 and 1986 (aged 16–21 years); the odds ratios were 0.42 (95% CI: 0.33–0.53), 0.42 (95% CI: 0.34–0.52), 0.77 (95% CI: 0.63–0.94), and 0.56 (95% CI: 0.47–0.75), respectively. By contrast, people born before 1952 (aged over 50 years) were more likely to test positive than the youngest cohort; the odds ratio was 2.19 (95% CI: 1.78–2.69).

The JE-neutralizing antibody positive rates were 65%, 72%, and 76% for the urban, suburban, and rural groups, respectively. When gender and birth cohort were adjusted for, compared with people living in urban areas, people living in rural areas had an odds ratio of 1.61 (95% CI: 1.31–1.98) for testing positive to JE-neutralizing antibodies, whereas those living in suburban areas had an odds ratio of 1.52 (95% CI: 1.34–1.74) for testing positive to JE-neutralizing antibodies.

## Discussion

The JE vaccination campaign in Taiwan has been running for more than 40 years, and the continual rescheduling of the immunization policy has resulted in various birth cohorts receiving distinct doses of the JE vaccine. This study revealed that prior to 1990, the age-group-specific incidence was higher among people <30 years of age than among older people but over the last decade appears to be similar or lower among people <30 years of age (Figure 3). These results might have been caused by the loss of antibodies, because they received the last dose long ago, received less than four doses of the vaccine, or received no vaccine at all.

Taiwan has been using an inactivated mouse brain Nakayama-NIH strain vaccine since 1968, except for the brief use of an inactivated freeze-dried Beijing strain vaccine in 1988 [13], [31]. According to the history of the public vaccination program, people born between 1963 and 1969 only received two primary doses, those born between 1970 and 1975 received three doses, and those born after 1975 received four doses, but those born before 1962 were never vaccinated because the immunization program had not yet been introduced [13]. Because the immunization program is national and compulsory, two-dose and three-dose schedules have achieved a coverage higher than 80%, and the coverage of the four-dose schedule has exceeded 95% [13].

The present JE neutralizing antibody study revealed that, among the people who were vaccinated (those born after 1963), the antibody positive rates decreased as people aged (Table 2). A possible explanation is that the vaccine-induced immunity wanes over time, regardless of the number of doses received. Regarding those who received four doses of the vaccine (the 1976–1980 and 1981–1986 cohorts), when serum samples were drawn in 2002, the antibody positive rates were 74% for the 1981–1986 cohort (10–15 years since the last booster dose) and 63% for the 1976–1980 cohort (16–20 years since the last booster dose). The odds ratio (OR) for cohort 1976–1980 compared to 1981–1986 (the reference) was 0.56; that is, the cohort 1976–1980 has approximately half odds of being positive than those of the cohort 1981–1986.The waning immunity phenomenon was less clear for the 1970–1975 and 1963–1969 cohorts, who received two or three doses (over 20 years since the last booster dose), because the positive rates were 54% for both cohorts. By contrast, among the people who were not vaccinated, the high seropositive rate of JE-neutralizing antibodies in the cohort born before 1952 was likely caused by a high frequency of early natural infection, as well as accumulated exposure, which induces a stronger immunological memory response than does vaccination [17], [18], [20], [32].

The results of a seroepidemiological study conducted in Taiwan from 1998 to 1999 by Tseng et al revealed that people with the lowest seropositive rate of JE-neutralizing antibodies in Kaohsiung and Pintung were those born between 1960 and 1969, and between 1960 and 1979, respectively [20]. In 1991, Shyu *et al* also determined that people born between 1962 and 1971 had the lowest seropositive rate of JE-neutralizing antibodies against JE [19]. The groups with the lowest seropositive rate of JE antibodies reported in the aforementioned studies differed from those identified in the present study. The studies mentioned were either regional or based on a small sample size, and thus not nationally representative, whereas this paper is based on a representative national sample [33]. A study that targeted the general Japanese population in 2004 determined that people aged 45–49 exhibited the lowest seropositive rate of JE antibodies [18], [32], which differed from the result obtained in Taiwan, despite their unclear birth cohort data.

The countries neighboring Taiwan have confronted similar problems of JE cases shifting from children to adults. Seventy-eight percent of confirmed JE cases in Japan between 1982 and 2004 occurred in people aged 40 or older, particularly in elderly people aged 60–69 years [18]. Similarly, 86.7% of the confirmed cases in Korea between 2007 and 2010 were adults aged above 40 years, particularly those between 40 and 49 years [17]. These statistics differ from those reported in most other countries worldwide, where children younger than 14 years account for 75% of confirmed JE cases [4]. Therefore, determining how to reduce the occurrence of adult cases of JE is critical for controlling and preventing JE in the future.

The incidence rates of JE have decreased considerably since 1971, mainly as a result of the implementation of childhood JE immunization programs that have successfully and quickly reduced the number of JE cases. The lowest point occurred between 1992 and 1997, with an average incidence rate of 0.066 per 100,000 people, and 14 confirmed cases every year. However there was a slight rise between 1998 and 2012, with an average incidence rate of 0.128 per 100,000 people, and 26 confirmed cases every year, which doubled the incidence rate of the period between 1992 and 1997. It remains unclear whether this rise is associated with changing testing methods or other factors. Japan reported fewer than 10 confirmed cases per year between 1992 and 2004 [18], and the average annual incidence rate of JE in Japan was only 0.004 per 100,000 people between 2000 and 2005 [34]. Korea reported a total of 45 confirmed cases between 2007 and 2010, with average annual incidence rates of 0.024 cases per 100,000 people [17]. After more than 40 years of JE vaccine policy implementation, the JE incidence in Taiwan remains higher than that in neighboring countries, thus, the long-term surveillance of JE is indispensable.

The present study revealed that the seropositive rate of JE-neutralizing antibodies was highest in rural areas and lowest in urban areas. This finding is consistent with a previous study in Taiwan [20], and indicates that the extent of natural infection is substantially affected by the degree of urbanization, and is likely to be caused by accumulated exposure. The JE virus is mainly transmitted by *Culex tritaeniorhynchus* and *Culex annulus* in Taiwan. However, effective control of these two outdoor mosquitoes is not possible, and various animals are hosts of JE [35]. Hence, JE is unlikely to disappear from the environment through human intervention [5].

The present study has some limitations. Because the survey of JE seroprevalence used specimens collected for the TwSHHH program in 2002, it is unclear if the program participants can represent the current seroprevalence. Obtaining their history of residence was also impossible, and thus, it was impossible to fully control the factor of urban versus rural residence. In addition, only 64% of the eligible subjects agreed to provide a blood sample. Although the gender and age ratio distributions did not differ considerably between the target study population and the total eligible participants, a potential selection bias cannot be dismissed. Another potential limitation is that Southern Taiwan has been threatened by outbreaks of DF every year since 1981 [36], and JE cases can only be confirmed when they meet certain criteria and exhibit a positive result in the laboratory. However, HI was used to detect JE antibodies before 1997; therefore, cross-reactivity might have occurred in reported cases in Southern Taiwan.

In conclusion, after more than 40 years of vaccination policy, the main age group of the confirmed JE cases shifted from young children to adults over 30 years of age during the last decade. This trend might be due to waning immunity or immunosenescence. Considering the poor prognosis and potential burden of this disease, JE remains a public health challenge in Taiwan. An adult immunization program and cost-effectiveness analyses require further study. The JE surveillance program requires strengthening to reduce the JE cases in adults.

## Supporting Information

Checklist S1 STROBE Checklist.
(PDF)
