# Supplementary material for: The Incidence of Japanese Encephalitis in Taiwan—A Population-Based Study
Source: PLoS Negl Trop Dis. 2014 Jul 24;8(7):e3030. doi: 10.1371/journal.pntd.0003030 (PMC4109885; doi:10.1371/journal.pntd.0003030)
Supplement: Checklist S1 — STROBE Checklist. (PDF) [file pntd.0003030.s001.pdf]

STROBE Statement—Checklist of items that should be included in reports of ***cross-sectional studies***

	Item No	Recommendation
**Title and abstract**	1	(*a*) Indicate the study's design with a commonly used term in the title or the abstract<br>☑ In the Methodology/Principal Findings
		(*b*) Provide in the abstract an informative and balanced summary of what was done and what was found<br>☑In the 'Methodology/Principal' and 'Conclusions/Significance Findings'
**Introduction**		
Background/rationale	2	Explain the scientific background and rationale for the investigation being reported<br>☑ The scientific background and rationale have included in paragraph 1-5 of the Introduction section.
Objectives	3	State specific objectives, including any prespecified hypotheses<br>☑ In the last paragraph of the Introduction section.
**Methods**		
Study design	4	Present key elements of study design early in the paper<br>☑In 'JE surveillance' of the Materials and Methods section.
Setting	5	Describe the setting, locations, and relevant dates, including periods of recruitment, exposure, follow-up, and data collection<br>☑ The data collection is depicted in 'The JE cases' and 'Seroprevalence of the JE-neutralizing antibody' of the Materials and Methods section.
Participants	6	(*a*) Give the eligibility criteria, and the sources and methods of selection of participants<br>☑ The eligibility criteria of participant is pictured at the Line 1-8 in Paragraph 2 of 'The JE cases' and at the Line 1-12 of 'Seroprevalence of the JE-neutralizing antibody' in the Materials and Methods section.
Variables	7	Clearly define all outcomes, exposures, predictors, potential confounders, and effect modifiers. Give diagnostic criteria, if applicable<br>☑ The urbanization is pictured at the Line 12-14 of 'Seroprevalence of the JE-neutralizing antibody' in the Materials and Methods section.
Data sources/ measurement	8*	For each variable of interest, give sources of data and details of methods of assessment (measurement). Describe comparability of assessment methods if there is more than one group<br>☑ The data sources are described at the Line 8-10 in Paragraph 2 of 'The JE cases' and at the Line 1-3 of 'Seroprevalence of the JE-neutralizing antibody' in the Materials and Methods section.
Bias	9	Describe any efforts to address potential sources of bias<br>☑ Not applicable for national mandatory surveillance data. Potential bias for the seroprevalence survey was addressed in the limitation section of the Discussion
Study size	10	Explain how the study size was arrived at<br>☑ It is in the Line 8-10 in Paragraph 2 of 'The JE cases' and at the Line 10-12 of 'Seroprevalence of the JE-neutralizing antibody' in the Materials and Methods section.
Quantitative variables	11	Explain how quantitative variables were handled in the analyses. If applicable, describe which groupings were chosen and why<br>☑ Measurements of the JE-neutralizing antibodies was described in section

		"Laboratory testing for the seroprevalence of JE".
Statistical methods	12	(*a*) Describe all statistical methods, including those used to control for confounding<br>☑ The statistical methods are described in 'Statistical analysis' at the Materials and Methods section. Logistic regression was used to adjust for covariates each other.
		(*b*) Describe any methods used to examine subgroups and interactions<br>☑ Not applicable.
		(*c*) Explain how missing data were addressed<br>☑ Not applicable for national mandatory surveillance data. Logistic regression for Seroprevalence used complete cases analysis.
		(*d*) If applicable, describe analytical methods taking account of sampling strategy<br>☑ Not applicable.
		(*e*) Describe any sensitivity analyses<br>☑ Not applicable.
**Results**		
Participants	13*	(a) Report numbers of individuals at each stage of study—eg numbers potentially eligible, examined for eligibility, confirmed eligible, included in the study, completing follow-up, and analysed<br>☑ In the Results
		(b) Give reasons for non-participation at each stage<br>☑ In the Results
		(c) Consider use of a flow diagram<br>☑ Not applicable.
Descriptive data	14*	(a) Give characteristics of study participants (eg demographic, clinical, social) and information on exposures and potential confounders<br>☑ In the Results
		(b) Indicate number of participants with missing data for each variable of interest<br>☑ Not applicable for national mandatory surveillance data. For seroprevalence survey, those with JE-neutralizing antibodies do not have missing data in demographic data.
Outcome data	15*	Report numbers of outcome events or summary measures<br>☑ They are included in the Results section.
Main results	16	(*a*) Give unadjusted estimates and, if applicable, confounder-adjusted estimates and their precision (eg, 95% confidence interval). Make clear which confounders were adjusted for and why they were included<br>☑They are depicted in 'JE among different birth cohorts' and 'Age distribution of confirmed JE cases' and 'Possible factors that affect the level of JE-neutralizing antibodies' in the Results section.
		(*b*) Report category boundaries when continuous variables were categorized<br>☑ The category boundaries of age in Results was reported.
		(*c*) If relevant, consider translating estimates of relative risk into absolute risk for a meaningful time period<br>☑ All absolute risks were described in rates per 100,000 persons.
Other analyses	17	Report other analyses done—eg analyses of subgroups and interactions, and sensitivity analyses<br>☑ Not applicable.
**Discussion**		

Key results	18	Summarise key results with reference to study objectives<br>☑It is at the Line 3 -10 in Paragraph 1 of the Discussion section.
Limitations	19	Discuss limitations of the study, taking into account sources of potential bias or imprecision. Discuss both direction and magnitude of any potential bias<br>☑It has incorporated in the last Paragraph of the Discussion section.
Interpretation	20	Give a cautious overall interpretation of results considering objectives, limitations, multiplicity of analyses, results from similar studies, and other relevant evidence<br>☑It is included in the end of the Discussion section.
Generalisability	21	Discuss the generalisability (external validity) of the study results<br>☑ In the discussion.
**Other information**		
Funding	22	Give the source of funding and the role of the funders for the present study and, if applicable, for the original study on which the present article is based<br>☑In the section Acknowledgement

*Give information separately for exposed and unexposed groups.

Note: An Explanation and Elaboration article discusses each checklist item and gives methodological background and published examples of transparent reporting. The STROBE checklist is best used in conjunction with this article (freely available on the Web sites of PLoS Medicine at http://www.plosmedicine.org/, Annals of Internal Medicine at http://www.annals.org/, and Epidemiology at http://www.epidem.com/). Information on the STROBE Initiative is available at www.strobe-statement.org.
